# Impact of Cross-Coupling Reactions in Drug Discovery and Development

**DOI:** 10.3390/molecules25153493

**Published:** 2020-07-31

**Authors:** Melissa J. Buskes, Maria-Jesus Blanco

**Affiliations:** Medicinal Chemistry. Sage Therapeutics, Inc. 215 First Street, Cambridge, MA 02142, USA; mjbuskes@gmail.com

**Keywords:** cross-coupling reactions, C-C bond forming reactions, C-Heteroatom bond forming reactions, palladium, clinical candidate, DNA-encoded libraries, cyclopeptides, allosteric modulators, PROTAC

## Abstract

Cross-coupling reactions have played a critical role enabling the rapid expansion of structure–activity relationships (SAR) during the drug discovery phase to identify a clinical candidate and facilitate subsequent drug development processes. The reliability and flexibility of this methodology have attracted great interest in the pharmaceutical industry, becoming one of the most used approaches from Lead Generation to Lead Optimization. In this mini-review, we present an overview of cross-coupling reaction applications to medicinal chemistry efforts, in particular the Suzuki–Miyaura and Buchwald–Hartwig cross-coupling reactions as a remarkable resource for the generation of carbon–carbon and carbon–heteroatom bonds. To further appreciate the impact of this methodology, the authors discuss some recent examples of clinical candidates that utilize key cross-coupling reactions in their large-scale synthetic process. Looking into future opportunities, the authors highlight the versatility of the cross-coupling reactions towards new chemical modalities like DNA-encoded libraries (DELs), new generation of peptides and cyclopeptides, allosteric modulators, and proteolysis targeting chimera (PROTAC) approaches.

## 1. Introduction

**Figure 1:**
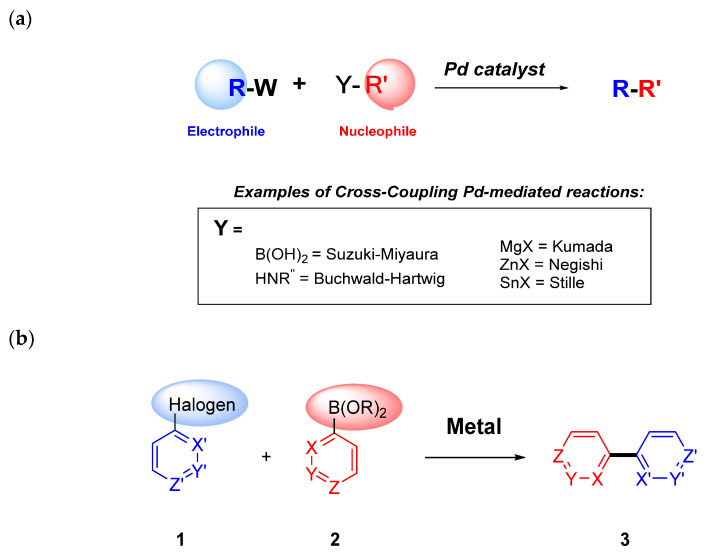
(**a**) Fundamentals of cross-coupling methodology; (**b**) example of Suzuki–Miyaura Csp^2^-Csp^2^ coupling.

Cross-coupling reactions have made an impressive impact in medicinal chemistry, and drug discovery and development for more than two decades. The success and popularity of this type of methodology comes from the fact that during the drug discovery phase, chemists are attracted to reactions that are reliable and reproducible. In drug discovery, medicinal chemists are designing and synthesizing novel compounds that could meet the criteria envisioned for the specific target product profile (TPP) to advance the eventual selected compound (referred to as Development Candidate) into clinical trials. During this period, medicinal chemists look for approaches to modify the structure in multiple ways to impart the corresponding pharmacological activity, pharmacokinetic and physicochemical attributes, in addition to a suitable safety profile, in general known as structure–activity relationships (SAR) and structure–property relationships (SPR). The cross-coupling methodology is a reliable and versatile approach that allows for the bond-formation of a sp^2^-hybridized aromatic halide (acting as an electrophile or **1**) with an organometallic (nucleophile or **2**) using a metal catalyst (Figure 1). In particular, palladium-catalyzed cross-coupling reactions have been demonstrated as an extraordinary resource and robust class of reactions [1] for the creation of carbon–carbon and carbon–heteroatom bonds in the pharmaceutical industry. In this review, we want to highlight the impactful applications of cross-coupling reactions in medicinal chemistry with a few recent examples from the field, with an emphasis on the Suzuki–Miyaura and Buchwald–Hartwig methodologies due to their versatility to generate carbon–carbon and carbon–heteroatom bonds and their prevalence as the two most common coupling reactions that contribute to medicinal chemistry [2] (For a broader scope of other cross-coupling reactions beyond the goals of this review, like Negishi [3,4], Kumada [5], Stille [6] and Chan-Lam [7], the reader is referred to additional reading.) Furthermore, we present specific recent cases for the identification of clinical candidates that incorporate cross-coupling reactions during their large-scale manufacturing process. As a forward outlook of these applications, we review several examples of broader utilization of cross-coupling reactions to new chemical modalities, such as DNA-encoded libraries (DELs), synthesis of novel cyclopeptides, allosteric modulators, and proteolysis targeting chimera (PROTAC) approaches.

The nomenclature for cross-coupling reactions is based on the type of nucleophile utilized. For example, using organoboranes as nucleophiles is referred to as Suzuki–Miyaura reactions (Figure 1), while using organozinc nucleophiles is known as the Negishi reaction.

French chemist Andre Job was the first person to combine organometallic reagents with catalysis in an effective fashion [8]. He reported mixing the Grignard PhMgBr with NiCl_2_ to incorporate CO, NO, C_2_H_4_, C_2_H_2_, or H_2_. Building on this initial discovery, in 1941, Kharasch published on metal-catalyzed homo-couplings of organomagnesium reagents [9]. Kharasch used catalytic amounts of FeCl_3_, CoCl_2_, MnCl_2_, or NiCl_2_ with Grignard reagents and alkyl or aryl halides for the homo-coupling reaction. After key contributions from Heck and others, Kochi [10] disclosed an FeCl_3_-catalyzed reaction between Csp^2^–Br electrophiles and Grignard reagents. In 1976, Negishi [11] demonstrated that other organometallic reagents could be used instead of Grignard reagents. Due to its bench stability and high reactivity, palladium was considered the preferred metal for cross-coupling reactions, and is now utilized in a routine basis across medicinal chemistry therapeutic areas.

## 2. C-C Reaction: Suzuki–Miyaura Reaction

**Figure 2:**
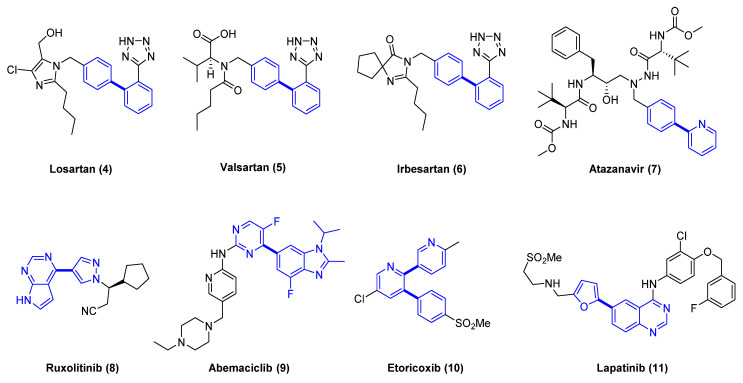
Examples of approved drugs where Suzuki–Miyaura coupling is employed to form critical carbon–carbon bonds with aromatic or heterocyclic groups.

In a recent study analyzing the most common reactions in medicinal chemistry [2], it was revealed that, in 2014, the Suzuki–Miyaura coupling was the second most utilized transformation after the amide bond formation. As a testimony to the impact of this reaction, it is possible to see a biphenyl moiety or aryl-heterocycle groups in a variety of approved drugs (Figure 2).

**Scheme 1:**
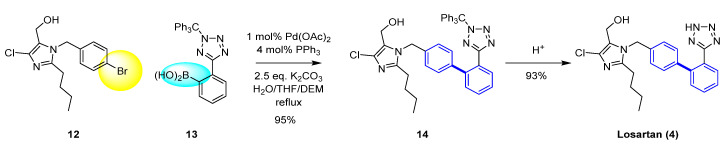
Example of the Suzuki–Miyaura coupling for the synthesis of Losartan (**4**).

As an example, the synthesis of Losartan (**4**) [12] is shown in Scheme 1 [13]. Losartan (**4**), one of the most prescribed medicines in the world, is the first angiotensin II receptor antagonist discovered by scientists at Dupont to treat high blood pressure. A key cross-coupling step in the synthetic route required further optimization in order to obtain a high yield. It was found that heating Pd(OAc)_2_ and triphenylphosphine in tetrahydrofuran (THF) led to the preparation of an active catalyst, instead of using tetrakis (triphenylphosphine) palladium, which was air-sensitive and not cost-effective for large-scale. The high temperature required for the coupling reaction led to the identification of a solvent system that mixed well with the water/base. A 1: 4 mixture of THF/Diethoxymethane (DEM) was identified as the most favorable for the catalyst preparation, affording a 95% yield.

A more recent example shown in Figure 2 is abemaciclib (**9**) [14], a CDK 4/6 inhibitor to treat advanced breast cancer with specific mutations HR+/HER2−, approved by the U.S. Food and Drug Administration (FDA) in 2017. The synthesis of abemaciclib (**9**) [15] incorporates a Suzuki cross-coupling reaction using a boronic ester followed by a palladium-mediated Buchwald–Hartwig amination to form the C-N bond with the pyrimidine ring.

The broad scope of the reaction to generate Csp^2^-Csp^2^ bonds, non-toxicity, tolerance toward varied functional groups, air- and moisture-stable properties, and straightforward synthesis made the Suzuki–Miyaura the top transformation of choice for SAR exploration in drug discovery. The research investment, mainly by academics, in developing new ligands and reaction conditions facilitated the synthetic feasibility and applicability to a large number of medicinal chemistry projects. This is one of the critical factors on how organic chemistry influences medicinal chemistry [16], helping to reduce the cycle times of the fundamental hypothesis, synthesis, and testing iterative processes in drug discovery.

The appealing use of Suzuki–Miyaura coupling led to multiple development candidates with these biphenyl groups. However, those biphenyls or “flat sp^2^-sp^2^ biaryls” introduced significant challenges in the developability [17] of this new chemical space. The molecules tended to be more lipophilic, with “flat” structure lattice resulting in high melting points and poor solubility. These developability issues created significant attrition [18] in clinical development. In a seminal paper, Humblet [19] and colleagues unveiled the concept of fraction sp^3^ (Fsp^3^, defined as number of sp^3^ hybridized carbons/total carbon count) as a measure of molecular complexity and established its correlation with the probability of technical success for compounds to transition from drug discovery to clinical testing and eventually becoming drugs. The higher the Fsp^3^ value, the higher probability of advancing the discovery candidate compound further into drug development, with approved small-molecule drugs having an average Fsp^3^ value of 0.47 [20]. This study marked the start of a paradigm shift in drug discovery towards increasing the three dimensionality (3D) of the molecules to minimize pharmaceutical development issues and increase the opportunities in clinical development.

**Figure 3:**
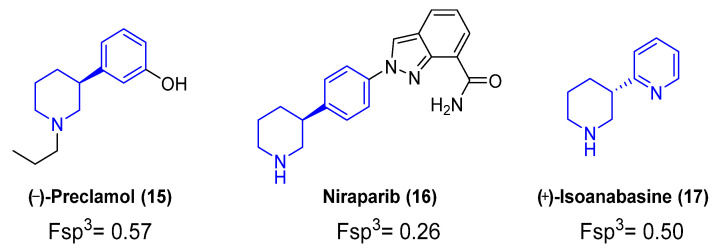
Clinical candidates synthesized via asymmetric Suzuki–Miyaura coupling reactions.

The development of synthetic methodology has evolved [21] to meet the need for preparing 3D-type molecules, including rhodium-catalyzed asymmetric Suzuki–Miyaura reaction with aryls, vinyls, heteroaryls, and heterocycles. Application of the asymmetric sp^2^-sp^3^ Suzuki–Miyaura methodology [22] enabled the synthesis of clinical candidates preclamol (**15**, a dopamine D2 receptor partial agonist studied for the treatment of schizophrenia), niraparib (**16**, MK−4827, a 2017 approved poly-(ADP ribose) polymerase (PARP) inhibitor indicated for ovarian cancer), and natural product isoanabasine (**17**), which are all presented in Figure 3 with their corresponding Fsp^3^ values calculated using SwissADME [23] online tool.

**Figure 4:**
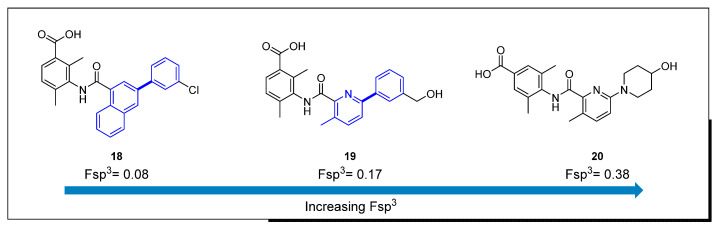
EP4 antagonists and their corresponding Fsp^3^ values.

**Scheme 2:**
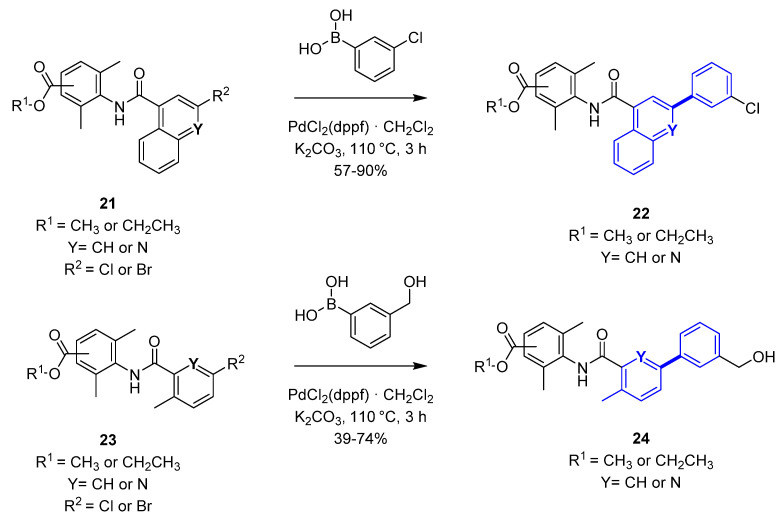
Synthetic approach for the preparation of EP4 antagonists.

An example of the trend to increase Fsp^3^ during the Lead Optimization phase, leveraging cross-coupling reactions in drug design, comes from the evolution of the SAR approach for novel E-prostanoid receptor 4 (EP4) antagonists (Figure 4) [24,25] that eventually led to the discovery of LY3127760 [26]. An initial lead (naphthalene derivative, **18**) [27] was identified through an early medicinal chemistry effort, possessing modest human whole blood activity (hWB, IC_50_ = 243 nM) and low Fsp^3^. The central core was further explored to increase ligand efficiency and sp^3^ character. Replacement of the naphthalene core with 2-methyl benzene or 3-methyl-pyridine led to the discovery of **19** (hWB IC_50_ = 39 nM, Fsp^3^ = 0.17), a compound with suitable pharmacokinetic profile to test in vivo. Additional SAR optimization to augment Fsp^3^ led to clinical candidate **20** with an Fsp^3^ of 0.38. The crucial synthetic steps for the preparation of compounds **18** and **19** are Pd-mediated cross-coupling reactions (Scheme 2). Suzuki–Miyaura coupling of quinoline or naphthalene (**21**) with 3-chloro-phenyl-boronic acid using PdCl_2_(dppf)·CH_2_Cl_2_ and potassium carbonate led to the corresponding ester derivatives (**22**) as penultimate compounds in the synthesis. This approach led to a route that enabled a rapid exploration of analogs for SAR optimization. In a similar fashion, Suzuki cross-coupling of 3-(hydroxymethyl)-phenyl-boronic acid with halogen-substituted phenyl or pyridine derivatives (**23**), using PdCl_2_(dppf)·CH_2_Cl_2_, provided the bi-aryl-ester analogs **24**.

## 3. C-N Reaction: Buchwald–Hartwig Reaction

Another breakthrough cross-coupling transformation in drug discovery is the Buchwald–Hartwig reaction that enables the C–N bond formation [28]. The introduction of nitrogen atoms is a fundamental approach in drug design [29] to modulate the lipophilicity of the molecules and their attributes like pharmacokinetic profile, brain penetration, solubility, permeability, etc. The versatile scope of the reaction resulted in it being one of the most used reactions in medicinal chemistry [30].

**Figure 5:**
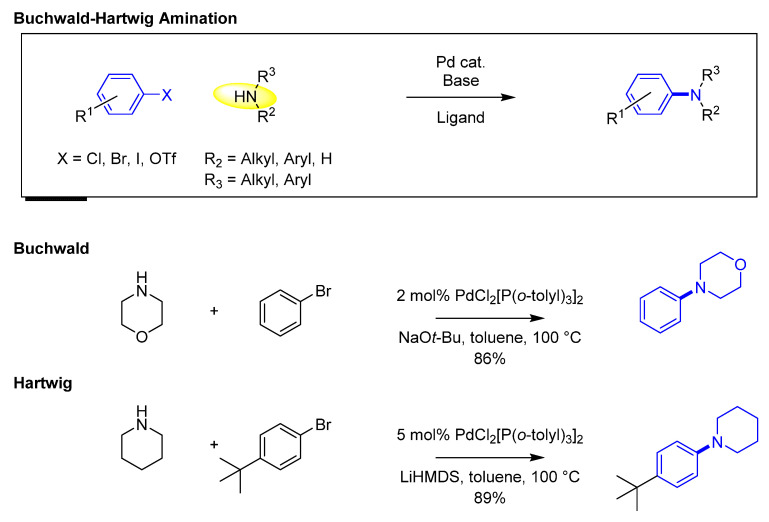
Buchwald-Hartwig Amination.

The major contributions by Stephen Buchwald and John Hartwig towards palladium-catalyzed cross-coupling reactions of amines with aryl halides between 1994 and 2000’s led to the methodology being known as Buchwald–Hartwig amination (Figure 5). The cross-coupling reaction fulfilled a need in medicinal chemistry of effectively generating aromatic C–N bonds, as other traditional methods, such as nucleophilic substitution, reductive amination, and amide coupling, as a few examples, have limited applicability.

**Figure 6:**
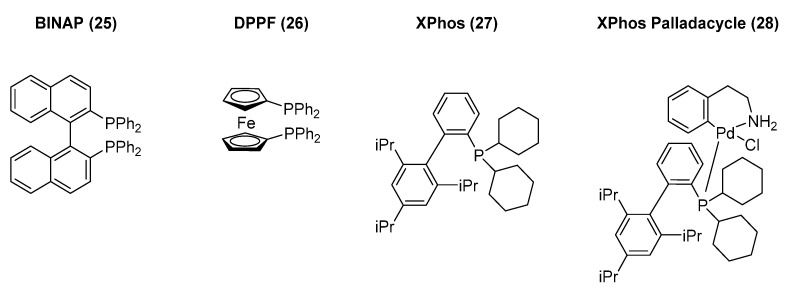
Key ligands used in palladium-mediated cross-coupling reactions.

The investment into developing structurally distinct ligands led to multiple novel catalyst systems that enabled an increase in the scope of the reaction to hindered amines or moderately reactive ones. The catalysts diphenylphosphinobinapthyl (BINAP, **25**) and diphenylphosphinoferrocene (DPPF, **26**) (Figure 6) enabled the initial extension to primary amines and the application to use aryl iodides or triflates. One of the advantages of those bidentate ligands was the prevention of generating the palladium iodide dimer after the oxidative addition step, thus accelerating the reaction and lowering the amount of palladium required. Large, bulky, sterically hindered tri- and di-alkyl phosphine ligands have demonstrated their ability to be resourceful catalysts, enabling the coupling of a vast variety of amines (primary, secondary, electron deficient, heterocyclic, etc.) with aryl or heteroaryl chlorides, bromides, iodides, and triflates. Further investigation into reaction conditions using different bases (e.g., hydroxide, carbonate, and phosphate bases) allowed for the development of stronger or milder conditions depending on the functional moieties in the substrates. The Buchwald group has focused mainly on generating a large variety of biaryl phosphine ligands, whereas the Hartwig group has directed their research towards ferrocene-derived phosphine ligands. In the last two decades, the interest in these transformations has led to general approaches and reliable protocols that are considered essential for SAR exploration and medicinal chemistry efforts.

**Figure 7:**
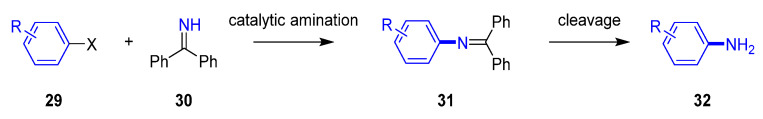
General strategy of using benzophenone imine as an ammonia equivalent.

One of the biggest challenges for the Buchwald–Hartwig reaction is the use of ammonia as a coupling partner, as it can bind tightly to palladium complexes. The generation of primary aniline derivatives is often found in the synthetic routes for more elaborated medicinal chemistry analogs, as it is a versatile precursor for the synthesis of *n*-based heterocycles or further elaboration of the aniline to amide derivatives. The direct use of ammonia gas as a coupling partner has significant limitations from a safety and handling perspective, even generating polyarylated side products. To overcome this issue, it has been disclosed an approach using ammonia equivalents [31]. This strategy is based on the utilization of benzophenone imine (**30)** [32] or silylamide [33] as coupling partners in the palladium-catalyzed cross-coupling reaction, and subsequent hydrolysis to yield the corresponding primary aniline (**32**, Figure 7). This method of employing ammonia surrogates is a milder synthetic alternative in comparison to other well-known routes like nitration and corresponding reduction steps.

**Scheme 3:**
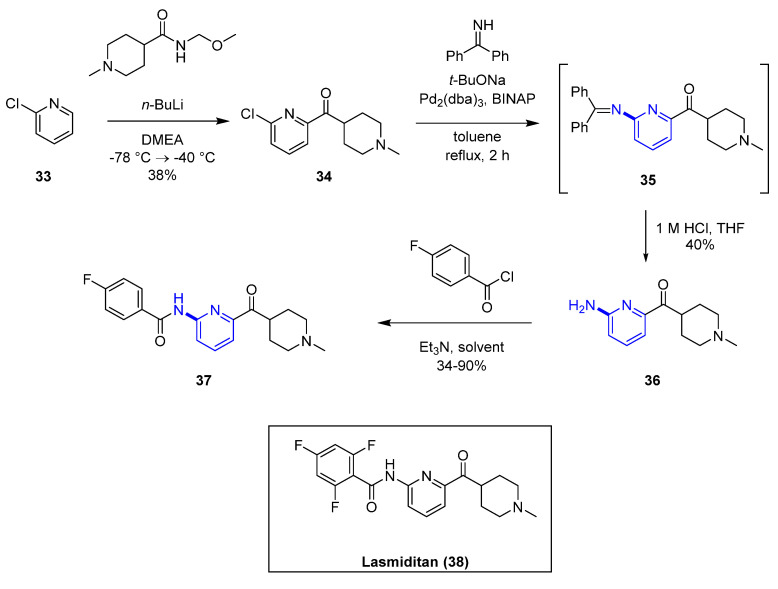
Example of 5-HT_1F_ receptor analogs.

Palladium-catalyzed amination using a benzophenone imine is widely used in medicinal chemistry. As an example, this methodology was applied in the SAR exploration for selective 5-HT_1F_ receptor agonists [34] (Scheme 3) that eventually led to the 2019 approved drug for acute migraine, Lasmiditan (**38**) [35]. Treatment of the key precursor halo-pyridinyl-(1-methyl-piperidin-4-yl)-methanone (**34**) with benzophenone imine, Pd_2_(dba)_3_, BINAP in the presence of *t*BuONa, led to the corresponding (6-((diphenylmethylene)amino)pyridin-2-yl) (1-methylpiperidin-4-yl) methanone derivative (**35**) in situ, that was hydrolyzed with hydrochloric acid to afford the desired amino-pyridine **36**. Acylation with substituted benzoyl chloride led to compound **37**, a selective 5-HT_1F_ agonist displaying >90-fold in vitro selectivity over 5-HT_1A_ and 5-HT_1D_, minimizing potential side effects of broad activation of those serotonin receptors.

**Figure 8:**
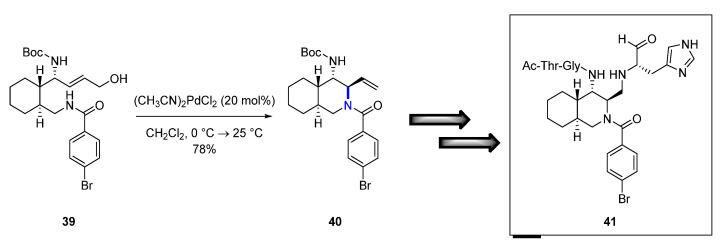
Cross-coupling reaction in the synthesis of SARS 3CLPRO inhibitors.

Cross-coupling reactions have been employed in medicinal chemistry campaigns across therapeutic areas, including the development of novel antiviral compounds as in the next example. In 2019, Akaji and colleagues [36] reported their efforts to evaluate decahydroisoquinoline inhibitors as severe acute respiratory syndrome (SARS) 3-chymotrypsin-like (3CL) protease inhibitors. The campaign aimed for the treatment of the SARS caused by a beta coronavirus (CoV) back in 2003, and so prevalent unfortunately in 2020 [37]. The CoV contains an RNA genome that encodes two polyproteins with a 3CL cysteine protease (SARS 3CLPRO). This protease is critical for viral replication and is not found in humans (or host cells), and therefore constitutes an attractive target for novel antivirals as there is no current available treatment. Applying structure-based drug design and to further optimize potency of a previous decahydroisoquinoline derivative, the researchers hypothesized that the 4-position carbon of the decahydroisoquinoline is the ideal place to incorporate a non-prime substituent (Figure 8). A key step of the synthesis is the Pd-catalyzed diastereoselective cyclization to generate the appropriate substituted decahydroisoquinoline **40**. Treatment of compound **39** with bis-acetonitrile palladium chloride led to the desired crucial intermediate **40** in 78% yield. This approach resulted in four analogs that were tested for their inhibitory activity against SARS 3CLPRO and the discovery of compound **41** as the most potent analog among them.

## 4. Cross-Coupling Reactions and Process Chemistry

Palladium cross-coupling reactions have a profound impact in the drug discovery phase, enabling the rapid functionalization of key precursors and exploration of SAR, as we have reviewed several cases in previous sections. The impact of this synthetic methodology is also appreciated in process chemistry during the large scale-up for clinical studies of development candidates. In particular, in process chemistry there are examples of specific cross-coupling reactions that are yield-efficient on a kilogram scale with cost-effective low catalyst loading. Purification of the desired product on a large-scale might become more complex, and the development of new technologies like metal scavenging or fixed-bed absorption processes enable the isolation of the desired cross-coupling product with minimal levels of palladium residue (<1 ppm) [38] to meet guidelines from regulatory agencies for active pharmaceutical ingredients (API). In addition of new methodology to remove traces of palladium or other catalysts, there is a strong trend to develop more “green” or environmentally friendly approaches to minimize potential toxicity to the ecosystem. We refer the reader to additional resources [39,40] for in depth focus of green chemistry metrics for development of pharmaceutics.

**Figure 9:**
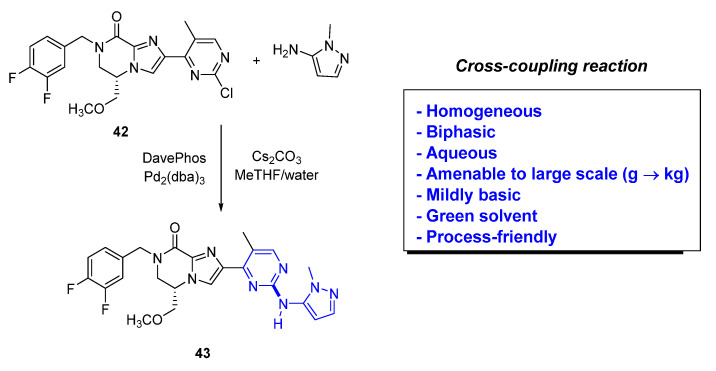
Strategies which enable “green chemistry” approach toward Buchwald–Hartwig amination.

As an example, exploration into these alternate “green” [41] conditions (Figure 9) led to the use of a biphasic reaction medium composed of 2-methyltetrahydrofuran (MeTHF) and water, to assist solubilizing the inorganic base. This helped overcome a common issue encountered with scalability of Buchwald–Hartwig aminations.

**Scheme 4:**
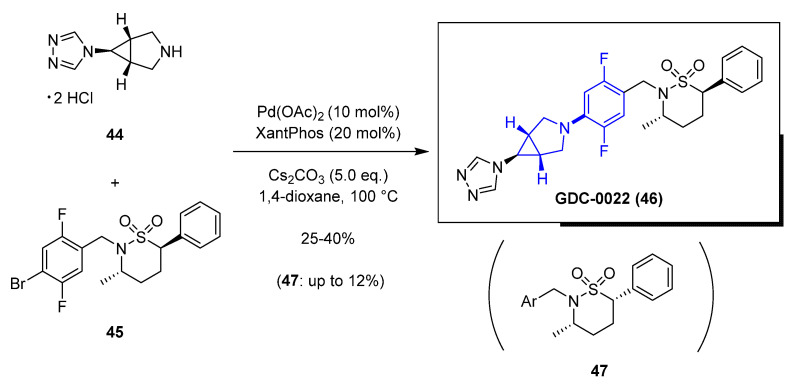
Initial results for the Buchwald–Hartwig C–N cross-coupling last step synthesis of GDC-0022 (**46**).

In this section, we highlight several recent examples of application of cross-coupling reactions to process chemistry. In 2020, the Genentech process group [42] reported the research to overcome a difficult palladium-catalyzed C–N bond formation for the scale-up synthesis of their clinical candidate GDC−0022 (**46**), an inhibitor of the nuclear hormone retinoic acid-related orphan receptor γ (RORγ). The clinical compound contains a sultam moiety with two chiral centers, and the coupling of the bromide-derivative containing the sultam is needed to proceed under mild conditions to ensure chiral integrity. The team explored the initial route developed by the discovery group that included a late-stage Buchwald–Hartwig C–N coupling. Upon initial exploration of that sequence, the process group found out that even 10 mol% of Pd(OAc)_2_ led to poor overall yields and produced epimerization leading to the undesired cis-(3*S*, 6*S*) diastereomer **47** (Scheme 4). Another challenge was the hydrodehalogenation by-products of the reagents. Initial attempts to scale-up to 150 g led to poor conversion (25% yield) and substantial amounts of the corresponding epimer **47** (12%). To make matters worse, the separation of GDC−0022 (**46**) vs. **47** was not easy and required expensive and time-consuming chiral supercritical fluid chromatography (SFC) methods. This approach was not suitable for a kilogram development campaign. The team used a creative multiparameter optimization effort on a microscale to screen ~300 different Pd-cross-coupling conditions varying the solvent, using different bases (K_3_PO_4_ or K_2_CO_3_) to minimize the risk for epimerization, and Pd(OAc)_2_ with mono- and bidentate phosphine ligands (XantPhos, XPhos, RuPhos, DPEPhos, DPPF, and BrettPhos). Based on this exploration, the combination of XantPhos and Pd(OAc)_2_ using K_3_PO_4_ in 1,4-dioxane demonstrated to be superior and was applied to early development manufacturing work, successfully enabling the reaction on an 8 kg scale.

**Figure 10:**
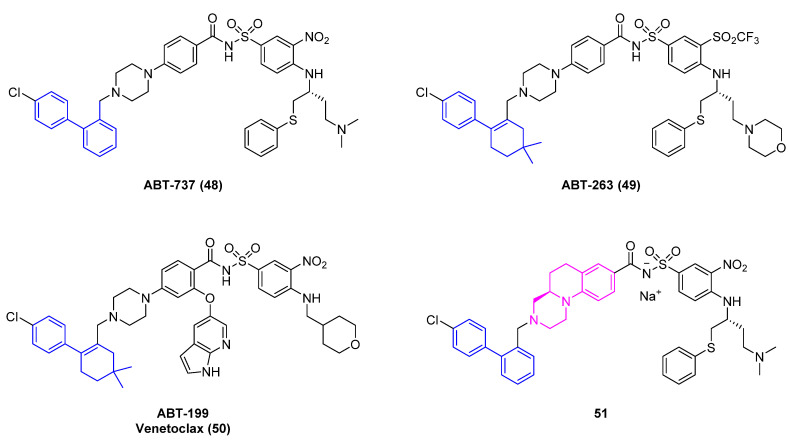
Bcl inhibitors ABT−737 (**48**), ABT−263 (**49**), Venetoclax (**50**), and **51**.

In the next example, the researchers applied a ligandless coupling approach using palladium supported on charcoal to synthesize a key biaryl moiety for a promising dual B cell lymphoma (Bcl)−2/-/Bcl-x_L_ inhibitor [43]. Antiapoptotic Bcl-2 family members have received great attention based on the clinical success of ABT−199 [44] (Venetoclax, **50**), which was approved by the FDA in 2016 for chronic lymphocytic leukemia [45]. However, Bcl inhibitors of this chemical class, for example, ABT−737 (**48**) and ABT−263 (**49**) (Figure 10), are large molecules with potential developability issues like low solubility that can reduce oral absorption. Scientists at Servier identified compound **51** (Figure 10) as a tricyclic analog of ABT−737 (**48**), specifically designed to decrease planarity of the central core and minimize solubility issues.

**Scheme 5:**
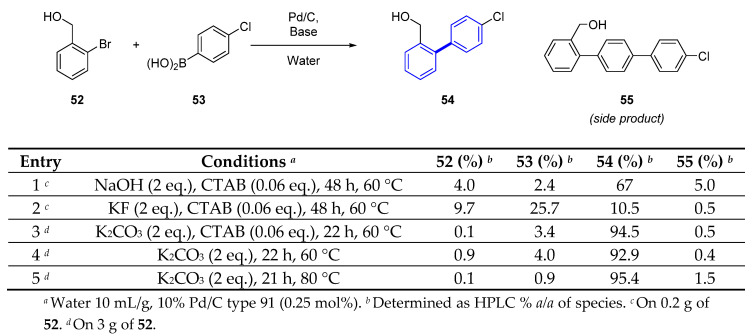
Reaction and screening conditions for Suzuki cross-coupling in water.

The authors tackle the challenge of a kilogram scale synthesis of **51**, and in particular the Suzuki coupling reaction of precursor **54** (Scheme 5), in an efficient manner. Although previously the reaction was carried out using Pd(OAc)_2_, the researchers wanted to develop a greener process exploring Pd/C type 91 as catalyst in water (Scheme 5). Based on their preliminary work, it was necessary to heat the reaction mixture, otherwise the reaction would stall. The use of K_2_CO_3_ as a base gave the best yields for compound **54**. The use of hexadecyl-trimethyl ammonium bromide (CTAB) did not further improve the conversion of the reaction. This is an interesting approach for large-scale synthesis in water for palladium mediated reactions.

**Figure 11:**
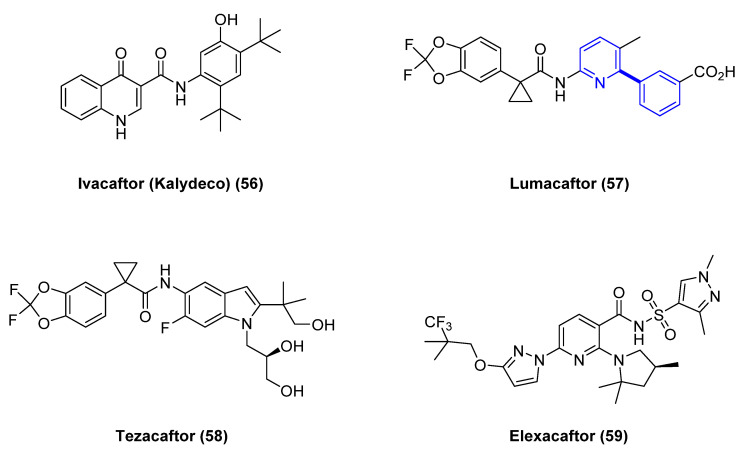
Cystic fibrosis transmembrane conductance regulator (CFTR)-modulator drugs for the treatment of cystic fibrosis.

Another impactful example of application of cross-coupling reactions to the development of meaningful medicines for patients comes from the synthesis of Lumacaftor (**57**) (Figure 11). Vertex has changed the landscape for cystic fibrosis with the recent approvals of Ivacaftor (**56**), Lumacaftor (**57**), Tezacaftor (**58**), and Elexacaftor (**59**) (Figure 11), and their corresponding combinations [46]. The rapid discovery and development of these four drugs since 2012 have been a game changer for science and patients [47]. A genetic mutation in the cystic fibrosis transmembrane conductance regulator (CFTR) [48] protein reduces its function leading to less chloride secretion with consequent increase of mucus in the airways, gastrointestinal tract, and other organs. These effects manifest clinically with gradual loss of pulmonary function, dietary deficits, and, eventually, could be fatal with respiratory failure. The most frequent genetic defect is the deletion of phenylalanine 508 (F508del). More than one CFTR modulator is needed to overcome the genetic defects in the CFTR protein. Positive allosteric modulators of the CFTR channel increase the probability of the open-channel state improving the ion gating through the channel. Small-molecule correctors such as Lumacaftor (**57**) behave as chaperones enabling protein folding and enhancing the trafficking of the CFTR proteins to the cell surface.

**Scheme 6:**
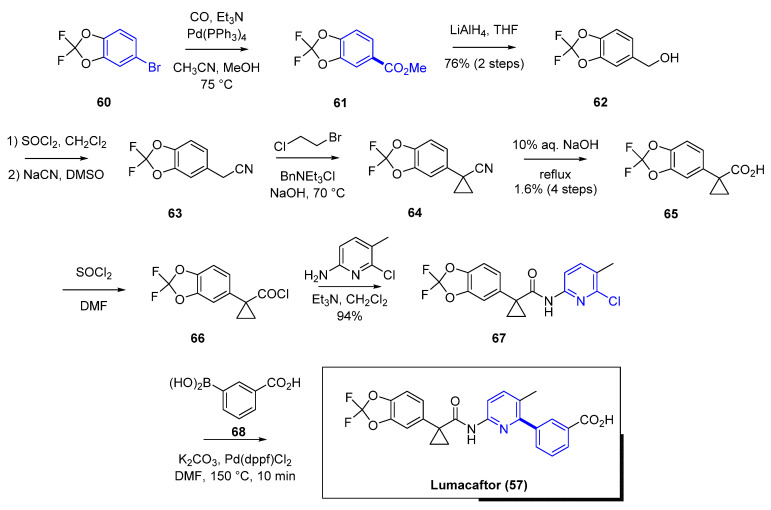
Synthesis of Lumacaftor (**57**).

**Scheme 7:**
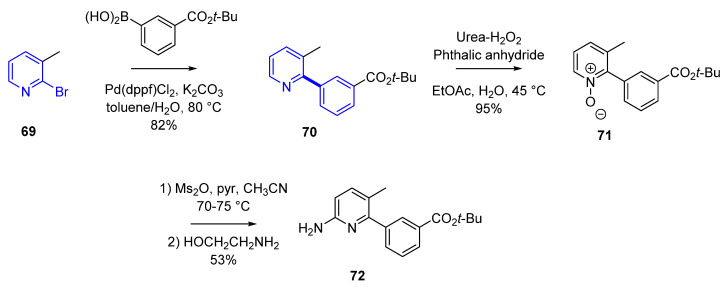
Alternate synthetic approaches for the preparation of Lumacaftor (**57**) precursors.

In the synthesis of Lumacaftor [49] (**57**, Scheme 6) there are two critical Pd-mediated cross-coupling reactions. For the assembly of the acid fragment **65**, the Pd-mediated carbonylation of the aromatic bromide **60** using Pd(PPh_3_)_4_, Et_3_N in methanol provided the corresponding methyl ester **61**. Further functionalization gave the desired acid **65** in 1.6% yield over the final four steps. After amide formation via an acid chloride to afford precursor **67**, the final step of the synthesis includes a Suzuki–Miyaura transformation. Cross-coupling using Pd(dppf)Cl_2_ in dimethylformamide (DMF) (at 150 °C, in the microwave for a short period of time) led to lumacaftor (**57**) (no yield was disclosed in the original synthesis) [50]. This initial synthesis was most likely used during the discovery phase to allow for an efficient synthesis of different analogs. However, once lumacaftor (**57**) was identified for clinical development, the synthesis was streamlined. Instead, the critical biaryl formation was performed first in the synthesis as displayed in Scheme 7. The Suzuki coupling of the boronic acid with 2-bromo-3-picoline **69** in toluene/water using Pd(dppf)Cl_2_ and K_2_CO_3_ at 80 °C provided a functionalized biaryl precursor **70** in an 82% yield.

## 5. Impact of Cross-Coupling Reactions on New Chemical Modalities

In the past few years, there has been an increasing interest in the medicinal chemistry community to explore additional chemical modalities beyond the traditional small molecules. These new chemical modalities [51] offer new opportunities to modulate targets and in particular, those challenging targets previously considered undruggable. As forward thinking, we want to offer an overview of the applications of cross-coupling reactions to these new chemical modalities.

### 5.1. Application of Cross-Coupling Reactions to DNA-Encoded Libraries (DELs)

Novel screening methodology using DNA-encoded libraries (DELs) [52] has been a focus of attention for multiple pharmaceutical companies as a way to enhance their high-throughput capabilities in early Lead Generation [53]. DNA-encoded synthesis allows a much broader evaluation of the traditional chemical space (~5-fold orders of magnitude) [54] coupled to miniaturization of high-affinity assays to test the different molecules. To design and synthesize DELs, reactions are required to be mainly feasible in aqueous conditions, and mild enough to preserve the DNA. Other considerations are assembly of building blocks, oligonucleotide conjugation, polymerase chain reaction (PCR) sequencing, and analysis of large data sets.

**Figure 12:**
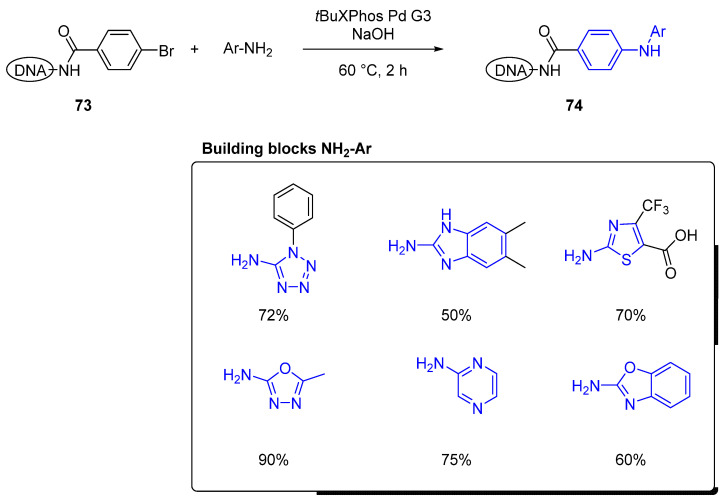
Examples of C–N cross-coupling reaction in DNA using various heteroaromatic amines. (Conversion was calculated by LCMS signal integration, not isolated yield.).

During the design process of a DEL library, the researcher needs to keep in consideration the types of reactions, specifically, they must be high yielding, broad scope, and primarily maintain the integrity of the DNA code. Many types of reactions have been able to adapt to mild and aqueous conditions required for DEL synthesis. For cross-coupling reactions, the Suzuki–Miyaura C–C coupling has been demonstrated in an example from the GlaxoSmithKline (GSK) group [55] towards hit identification of phosphoinositide 3-kinase α (PI3Kα) ligands, preparing a three-cycle library of over 3.5 million of diverse compounds. More recently, Torrado and colleagues [56] developed a methodology to tackle, for the first time, the C–N cross-coupling on DNA. Through a series of parallel screening conditions, the group identified mild and efficient reaction conditions for the *n*-arylation of anilines on-DNA aryl bromides. The strategy focused on identifying the palladium catalyst in multi-array aqueous conditions, using *t*BuOK as the base and temperatures between 50 and 80 °C, using first as a pilot aryl-iodides on DNA. After investigation of more than 12 different sources of palladium, the reaction only provided the desired product when *t*-butyl-XPhos Pd precatalyst G3 was used. Overall, a combination of *t*-butyl-XPhos Pd precatalyst G3 (15 eq.) using NaOH (300 eq.) as the base proved to be the most effective conditions for the generation of the C−N bond between conjugated DNA aryl iodides and aromatic amines at 30 °C. Based on these results, the group explored reaction conditions for the use of aryl bromides on DNA (**73**, Figure 12). Fortunately, minor adjustments in the reaction protocol, like increasing the number of equivalents of the aniline (from 80 eq. to 150 eq.) and the temperature to 60 °C, led to comparable catalytic abilities for DNA-conjugated aryl bromides, less reactive electrophiles, but more versatile building blocks. In Figure 12, it is shown further application of this methodology to heteroaryl cross-coupling reactions expanding the scope of the reaction for medicinal chemistry applications during hit identification. After applying this protocol to >850 structurally diverse (hetero)aromatic anilines and >450 aryl bromides conjugated to DNA, the authors reported the successful application of this new method to the production of a DEL during cycle 3.

### 5.2. Application of Cross-Coupling Reactions to Macrocycle and Cyclopeptides

Macrocycle and cyclopeptides are another chemical modality that it is currently attracting vast attention in medicinal chemistry [57] to modulate targets with larger binding pockets and provide novel mechanism of action. Strategies targeting traditional small molecules applying “rule-of-five” (Ro5) guidelines have been recognized to be unsuccessful against difficult targets, like protein–protein interactions, nucleic acid complexes, or antibacterial modalities. However, natural products have demonstrated to be effective at modulating such targets, directing a renewed interest on investigating underrepresented chemical scaffolds associated with natural products. Naturally derived cyclopeptides [58] offer advantages in affinity, selectivity, stability and druggability in comparison to linear peptides.

**Figure 13:**
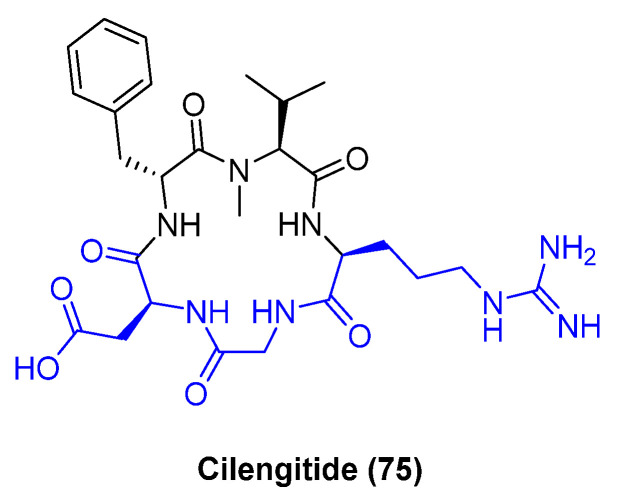
Structure of cyclopeptide cilengitide (**75**), RGD displayed in blue.

Using natural products as starting points, medicinal chemists have modified the core structure of the cyclopeptide to increase pharmacological activity and drug-like properties. There are examples of introducing biaryl systems within the cyclopeptide core like largazole [59] derivatives resulting in higher selectivity and potency. Recently Sewald [60] and colleagues developed a new methodology applying Suzuki−Miyaura cross-coupling reactions (in solution or on-resin) to introduce a biaryl moiety into an Arg-Gly-Asp (also known as RGD) cyclopeptide providing new SAR direction. Cilengitide (**75**, Figure 13) is a cyclic Arg-Gly-Asp peptide (displayed in blue) and potent αvβ3 and αvβ5 integrin inhibitor that received significant attention for its advanced clinical studies for glioblastoma. The critical role of integrins as intermediaries in a broad range of cancer cell activities resulted in multiple drug discovery efforts of this target for oncology.

**Scheme 8:**
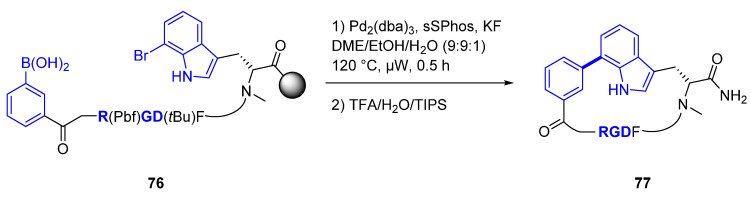
Suzuki-Miyaura Cross-Coupling for the synthesis of biaryl cyclopeptide **77**.

Sewald and his group used the Suzuki-Miyaura reaction as an approach towards side-to-tail cyclization incorporating the biaryl moiety. They incorporated bromo-tryptophan isomers within the peptide chain to enable an intramolecular palladium-mediated Suzuki-Miyaura cyclization reaction with a boronic acid in the other extreme of the peptide. The versatility of the Suzuki-Miyaura reaction allowed for the synthesis of multiple cyclopeptides and SAR evaluation. The initial conditions in solution were Na_2_PdCl_4_ with water-soluble sSPhos (sodium 2′-dicyclohexylphosphino-2,6-dimethoxy-1,1′-biphenyl-3-sulfonate hydrate). However, high dilution conditions (0.2 mM) to avoid intermolecular cross-coupling did not afford the desired cyclic peptide. Increasing the concentration of the cross-coupling reaction to 2.0 mM provided higher conversion without the dimerization by-product. Further optimization included increasing the reaction temperature to 100 °C. The cross-coupling reaction on-resin was adapted using Pd_2_(dba)_3_, potassium fluoride, and a solvent mixture of 1,2-dimethoxyethane (DME), ethanol (EtOH) and water (H_2_O) (Scheme 8). Using this approach more than 20 cyclopeptides were synthesized and evaluated in vitro. Interestingly, the connectivity between the aromatic group and the indole influenced the conformation of the cyclopeptide, the affinity and selectivity. As a result, cyclopeptide **77** was identified as a low nanomolar (5.4 nM) αvβ3 integrin with a connection 3 to 7– aromatic-indole respectively and high human plasma stability (t_1/2_ > 24 h).

### 5.3. Application of Cross-Coupling Reactions to Allosteric Modulators

Another interesting chemical modality that is currently receiving much attention is the design and synthesis of allosteric modulators [61,62,63]. This modality offers significant advantages over the development of orthosteric agonists, enabling much more fine-tuned modulation of the receptor only in the presence of the endogenous ligand. A successful allosteric modulator should be able to bind to a “remote” or secondary active site of the receptor and might produce changes in the conformation of the primary active site (or orthosteric site). Overall, this chemical modality provides ligands that offer suited compounds less prone to produce desensitization (like orthosteric agonists) and favorable toxicological profiles as allosteric modulators have a “ceiling” on the magnitude of their allosteric effect.

**Figure 14:**
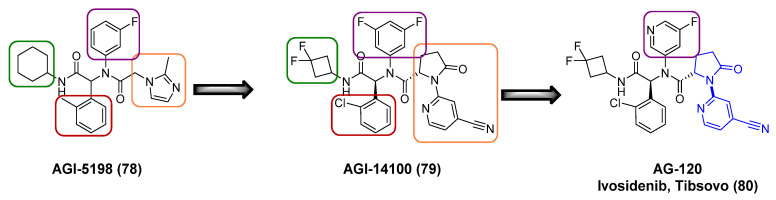
Evolution of the key breakthrough compounds for IDH1 inhibitors.

One recent example is the discovery of AG−120 [64] (ivosidenib) (**80**) (Figure 14), a highly specific, allosteric, reversible inhibitor of the isocitrate dehydrogenase−1 (IDH1) mutant enzyme (a mutation present in several types of cancer). The identification of IDH mutations among numerous cancer types has transformed the knowledge of oncogenesis and the opportunity for targeted therapeutics focus on small molecule inhibitors. Ivosidenib (**80**) was approved in the United States in 2018 for the treatment of relapsed or refractory acute myeloid leukemia (AML) for patients with the IDH1 mutation, followed by a 2019 FDA-approval for patients susceptible to the IDH1 mutation and upon first diagnosis.

An initial prototype mutant IDH1 inhibitor AGI−5198 (**78**, Figure 14) was identified from a high-throughput screen. Although AGI−5198 (**78**) showed a strong tumor inhibition in preclinical models, the poor pharmacokinetic profile (high clearance) prevented its further advancement into clinical studies. Medicinal chemistry approaches toward decreasing clearance rates by blocking metabolism were undertaken by incorporating fluorinated cycloalkyl groups (green box, Figure 14) and replacing the imidazole in the right portion of the molecule (orange box), which led to the discovery of AGI−14100 (**79**, Figure 14). AGI−14100 (**79**) exhibited high in vitro potency for IDH1 and suitable metabolic stability; however, further evaluation for the human pregnane X receptor (hPXR) indicated that the compound was a strong inducer of the cytochrome P450 (CYP) 3A4. Introduction of additional polarity in the central core of the molecule to minimize CYP induction, led to the replacement of the 3,5-di-fluoro-phenyl by a 5-fluoro-pyridine (purple box). The resulting compound, AG−120 (**80**), had the appropriate combination of in vitro potency and pharmacokinetic profile with reduced hPXR activation.

**Scheme 9:**
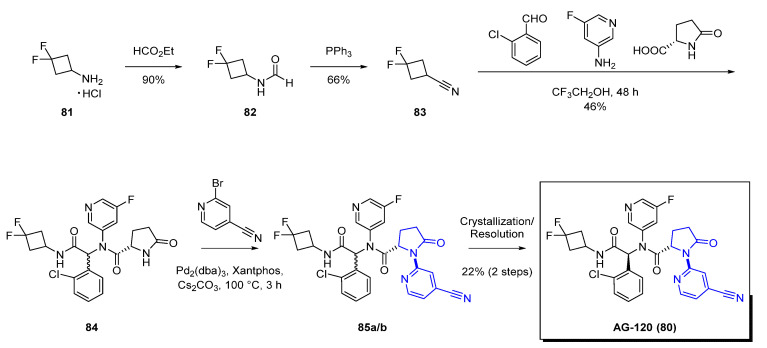
Synthesis of AG-120 (**80**).

The synthesis of AG−120 (**80**) is shown in Scheme 9. After preparing the intermediate **83** using standard literature procedures, the Agios group used a Ugi four-component reaction to prepare the key precursor **84** as a racemate in a 46% yield. Palladium-mediated cross-coupling reaction using Pd_2_(dba)_3_, Xantphos as the catalyst, and Cs_2_CO_3_ as the base with 2-bromo-isonicotinonitrile led to a diastereomeric mixture **85a/b**. Furthermore, after crystallization, the resulting mixture was submitted for chiral resolution giving the enantiomerically pure compound AG−120 (**80**). As the authors noted, the synthetic route was robust enough to be scalable to >100 g to enable the preclinical pharmacology and toxicology studies.

### 5.4. Application of Cross-Coupling Reactions in Proteolysis Targeting Chimeras (PROTACs)

Targeted protein degradation, in particular PROTACs, has attracted the attention of many academic institutions and pharmaceutical companies as a novel therapeutic approach in drug discovery [65,66]. This emerging technology has the ability to target the “undruggable” proteome, a limitation of traditional drugs [67,68]. These molecules are designed to enable ubiquitination of the target protein of interest, ultimately flagging it for degradation by the proteasome. PROTAC molecules possess a heterobifunctional structure that contains three chemical elements: a ligand that binds the desired target protein, a moiety that binds a specific E3 ligase, and a linker between the two. There are advantages of this strategy for drug development as maintaining a high level of drug dosing is not required as these degraders are catalytic in their mode of action. PROTACs have become a new chemical modality [51] with some companies like Arvinas being pioneers [69] and many other pharmaceutical companies focusing on this approach. As such, we have witnessed a revolution in this field with multiple publications across therapeutic areas. For the synthesis of PROTACs, we are observing different methodologies to synthesize the ligand that binds to the receptor and publications are emerging incorporating the application of cross-coupling reactions [70,71,72]. Among them, we have selected a specific example below where the use of Pd-mediated cross-coupling reaction connecting the ligand to the PROTAC linker is essential.

GSK focused their attention on targeting Interleukin−1 receptor-associated kinase 4 (IRAK4) degradation utilizing the small molecule PROTAC approach [73]. The effort aimed to target both IRAK4′s kinase and scaffolding protein functionality, currently unachievable by small molecule inhibitors. This strategy has the potential to have a greater therapeutic benefit and could lead to new therapeutic opportunities in the treatment of autoimmune, inflammatory, and cancer diseases.

**Scheme 10:**
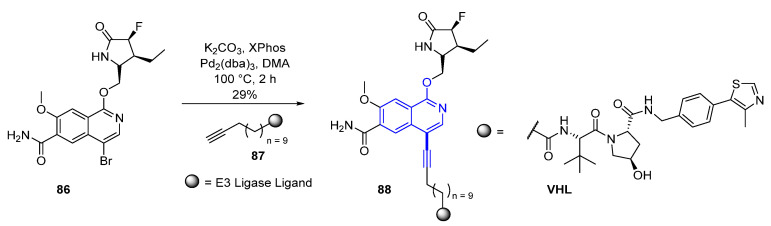
Synthesis of IRAK4 PROTACs linking E3 ligase ligand and IRAK4 ligand.

**Figure 15:**
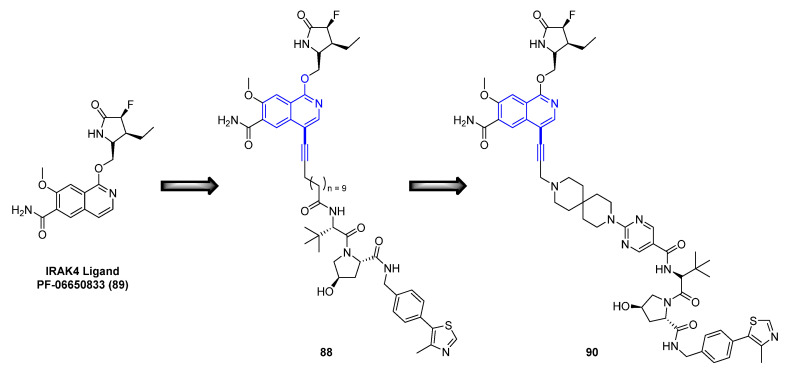
Evolution of small molecule IRAK4 ligand, PF−06650833 (**89**), to PROTAC degrader molecules **88** and **90**.

Efforts began by preparing IRAK4 degrader compounds utilizing a modified analog (not displayed) of the known IRAK4 kinase inhibitor PF−06650833 (**89**) (Figure 15) [74], in combination with either the von Hippel–Lindau (VHL), Cereblon (CRBN), or Inhibitor of Apoptosis (IAP) E3 ligase ligand attached via a linker moiety designed *in silico*. Rapid production and subsequent evaluation of these analogs’ degrader capabilities were facilitated by the development of a synthetic strategy, utilizing cross-coupling capabilities that productively enabled variations of the E3 ligase ligand and linker. This approach employed a Sonogashira coupling, attaching the IRAK4 ligand analog to the desired E3 ligase ligand and linker moiety (general reaction conditions displayed in Scheme 10). The conditions for the coupling reaction utilized Pd_2_(dba)_3_ as the palladium source in the presence of XPhos, potassium carbonate as the base in *N*,*N*-dimethylacetamide (DMA). Although variations in catalyst, reagent loadings, and reaction times were required, this methodology proved to be a robust route for the rapid production of analogs.

After several iterations of IRAK4 PROTAC design, the fully functionalized, 4-bromo derivative of PF−06650833 (**86**) was installed onto the identified IRAK4 PROTAC VHL linked warhead (**87**), utilizing the established coupling conditions previously detailed, resulting in the discovery of compound **88** (Scheme 10/Figure 15). Compound **88** was found to display potent degradation with a half-maximal degradation concentration (DC_50_) in peripheral blood mononuclear cells (PBMC) cells of 259 nM. Further optimization efforts focused around modifying the polarity and flexibility of the linker moiety. This exploration was expedited by the previous screening cross-coupling reaction conditions and led to a more rigid, polar spirocyclic pyrimidine linker, compound **90** (Figure 15), which displayed a further improvement in potency (DC_50_ of 151 nM in PBMC cells). Additional studies revealed that the degradation took place in a proteasome dependent manner.

Further studies of the compound elucidated that not all IRAK4 functions were inhibited despite IRAK4 degradation, highlighting the need for a more thorough understanding of the target itself. Regardless, these compounds achieved potent intracellular degradation of IRAK4 as proof of concept. This strategy showed promise in the identification of novel therapeutic indications.

## 6. Conclusions

In this overview of the applications of cross-coupling reactions to medicinal chemistry and drug discovery, we have presented a selection of recent examples from a diverse set of therapeutic indications including migraine, oncology, pain, antiviral, autoimmune diseases, and cystic fibrosis. Cross-coupling reactions are used nowadays as a common and reliable approach to build SAR assessments. Furthermore, the application of cross-coupling methodology has become more frequent for the large scale-up synthesis in the development phase, as illustrated in this manuscript. Current advances are directed to develop “greener” conditions for process cross-coupling transformations. New chemical modalities are an emerging area of interest in drug discovery, where researchers are looking for novel chemical space to modulate challenging targets. We thought it would be important to showcase how cross-coupling methodology can be instrumental in identifying novel starting points for Lead Generation campaigns through DELs, macrocycles and cyclopeptides, allosteric approaches, as well as PROTACs efforts. At the core of this attention, lies the flexibility and reproducibility of the reaction conditions, to enable a vast set of transformations. We are envisioning a bright future for further development in this field with newer and milder cross-coupling conditions to enable reactions even in physiological relevant environments.

## Abbreviations

5-HT	5-hydroxytryptamine or serotonin
API	Active pharmaceutical ingredient
BINAP	diphenylphosphinobinapthyl
C-C	Carbon-Carbon
CFTR	cystic fibrosis transmembrane conductance regulator
C-N	Carbon-Nitrogen
CoV	CoronaVirus
CTAB	hexadecyl-trimethyl ammonium bromide
DC_50_	half maximal degradation concentration
DEL	DNA-encoded library
DNA	Deoxyribonucleic acid
DPPF	diphenylphosphinoferrocene
EP4	E-prostanoid receptor 4
FDA	Food and Drug Administration
Fsp^3^	Fraction sp^3^
IC_50_	half maximal inhibitory concentration
IDH1	Isocitrate dehydrogenase−1
IRAK4	Interleukin−1 receptor-associated kinase 4
nM	nanoMolar
Pd	Palladium
ppm	parts per million
PROTACs	Proteolysis Targeting Chimeras
RGD	Arg-Gly-Asp amino acid sequence
Ro5	Rule of Five
SAR	Structure-Activity Relationships
SARS	severe acute respiratory syndrome
SPR	Structure-property Relationships
